# Risk of Ischaemic Stroke Varies With Antithrombotic Drugs Use in Proton Pump Inhibitor Users: A Self‐Controlled Case Series Study

**DOI:** 10.1002/pds.70219

**Published:** 2025-09-18

**Authors:** Min Fan, Joseph E. Blais, Ian C. K. Wong, Jesse Zhao, Ka Shing Cheung, Esther W. Y. Chan, Angel Y. S. Wong, Celine S. L. Chui

**Affiliations:** ^1^ Centre for Safe Medication Practice and Research, Department of Pharmacology and Pharmacy, Li Ka Shing Faculty of Medicine The University of Hong Kong Hong Kong Special Administration Region China; ^2^ Hong Kong Science Park, Hong Kong Science and Technology Park Hong Kong Special Administration Region China; ^3^ School of Public Health, LKS Faculty of Medicine The University of Hong Kong Hong Kong Special Administration Region China; ^4^ Department of Medicine, School of Clinical Medicine The University of Hong Kong, Queen Mary Hospital Hong Kong Special Administration Region China; ^5^ Department of Medicine The University of Hong Kong‐Shenzhen Hospital Shenzhen China; ^6^ Department of Non‐Communicable Disease Epidemiology, Faculty of Epidemiology and Population Health London School of Hygiene and Tropical Medicine London UK; ^7^ School of Nursing The University of Hong Kong Hong Kong Special Administration Region China

**Keywords:** anticoagulant, antiplatelet, proton pump inhibitors, self‐controlled case series study, stroke

## Abstract

**Background:**

The conflicting findings on the association between proton pump inhibitors (PPIs) and ischaemic stroke could be due to residual confounding. Self‐controlled case series (SCCS) can be used to avoid time‐invariant confounding. Additionally, different baseline risks of stroke should be considered, as some individuals may be prescribed PPIs for gastroprotection from bleeding with antithrombotic drugs.

**Methods:**

We identified adult patients with incident ischaemic stroke from 2003 to 2014 in Hong Kong and applied the modified SCCS. The exposure window was pre‐defined as Days 1–30, 31–60, 61–90, and 91 to the prescription end, since the PPI prescription. All other periods were referent windows. We estimated incidence rate ratios (IRR) and stratified them further using antithrombotic drugs.

**Results:**

A total of 18 170 patients were included. The IRRs for ischaemic stroke were 1.55 (95% CI: 1.00–2.42) during days 61 to 90, 1.51 (95% CI: 1.14–2.00) during days 91 to end, versus the referent window. There was no evidence of an increased risk in other risk windows versus the referent windows. In the stratified analysis, we observed an increased risk in people co‐prescribed PPIs with antithrombotic drugs in all risk periods, but no increased risks among those with PPI monotherapy versus the referent window.

**Conclusion:**

No evidence of a higher ischaemic stroke after monotherapy of PPI use. The increased risk of ischaemic stroke associated with PPIs could be due to their high baseline risk prescribed with antithrombotic drugs for primary prevention. Clinical monitoring of ischaemic stroke is recommended in these people.

**Summary**

- A new method shows no increased stroke with just PPIs, but a higher risk when PPIs are used with blood thinners.
- It highlights the importance of monitoring co‐prescribed users and the appropriate methodology used.

## 1 Introduction

Proton pump inhibitors (PPIs) are acid‐suppressing drugs for the treatment of peptic ulcers, gastro‐oesophageal reflux disease, and *Helicobacter pylori* infection [1, 2]. Despite their well‐tolerated safety profile, emerging observational data suggest a range of potential harmful effects of PPIs, including dementia [3, 4], gastric cancer [5], and cardiovascular events [6, 7, 8]. Recently, there has also been inconclusive evidence about PPIs and the risk of ischaemic stroke [9, 10]. The existing evidence suggests an ADMA‐mediated mechanism may explain the increased risk of ischemic stroke observed with PPI use [11, 12, 13, 14, 15, 16, 17, 18, 19]. Yet, the impact of PPIs on ADMA levels and the associated risk of ischemic stroke in humans remains unclear [20]. On the other hand, some suggest that PPIs can prevent ischaemic stroke through anti‐ *H. pylori* infections, which is a potential risk factor for ischaemic stroke [21, 22, 23]. However, our previous works did not find a lower risk of ischaemic stroke associated with *H. pylori* therapy containing PPIs [21, 24].

Adequately controlling confounders is essential for exploring the cardiovascular disease (CVD) risks after PPI use. For instance, the observed risk of myocardial infarction associated with PPI is likely due to confounding and protopathic bias [25]. For the association between PPIs and ischaemic stroke, PPIs are often prescribed with antithrombotic drugs for gastroprotection [26, 27], but the use of antithrombotic drugs is not fully accounted for in current literature [10, 28]. Furthermore, most previous analyses utilized conventional study designs, such as cohort and case–control studies, which are susceptible to unmeasured confounding. It is challenging to study the safety of PPIs due to differing health characteristics between users and non‐users. A previous cohort study shows that despite using high‐dimensional propensity scores, residual confounding remained when investigating the association between PPI use and poor health outcomes [29].

The self‐controlled case series (SCCS) is widely applied in pharmacoepidemiologic studies since it addresses time‐invariant confounding [25, 30, 31, 32]. In this study, we aimed to investigate the association between PPIs and ischaemic stroke using SCCS. Meeting key assumptions to obtain robust SCCS results is crucial [33, 34]. As the occurrence of an ischaemic stroke may influence subsequent exposure or the observation periods, which are key assumptions of SCCS, a modified instead of a standard SCCS model was applied. Besides, since the risk of ischemic stroke may differ based on antithrombotic drugs use for the PPI users, we conducted stratified analyses.

## 2 Methods

This study utilized a SCCS with a population‐based electronic health records database in Hong Kong. Given the potential violation of assumptions in the standard SCCS, we illustrated the assumption check plots and subsequently employed a modified SCCS approach. The study protocol was approved by the Institutional Review Board of the University of Hong Kong/Hospital Authority Hong Kong West Cluster (IRB reference number: UW 21‐197).

### 2.1 The SCCS Design

The SCCS design sets an observation period for each participant and then compares the risk of ischaemic stroke during the predefined exposure period (periods following PPI prescription) with the referent window (all other periods except the exposure window). By using each individual as their own control, SCCS inherently controls the time‐invariant confounding factors, for example, sex, genetic factors, etc.

There are some major assumptions for a standard SCCS (details in Supporting Information 1). The outcome of interest in this study might potentially violate event‐dependent exposure and event‐dependent observation assumptions. For instance, a patient with an ischaemic stroke episode might affect prescribers' decisions on the subsequent PPI prescription, which could violate the assumption of event‐dependent exposure. On the other hand, ischaemic stroke also increases mortality risk that would end the observation period prematurely, which could violate the assumption of event‐dependent observation. The assumption was assessed, shown in Supporting Information 1.

Therefore, instead of using a standard SCCS model, we addressed potential assumption violations by introducing a novel modified SCCS model, which simulates an extreme situation in which all exposures after the events are dismissed [33, 35]. However, the real dataset indeed observed some exposure after the events. Hence, event numbers in those exposure periods were adjusted to fulfill the counterfactuals. A previous study demonstrates the applicability of this method to investigate the safety of COVID‐19 vaccines [33], and it was widely applied on COVID‐19 vaccines [30, 33, 36, 37, 38].

### 2.2 Data Source

This study used electronic health records from the Clinical Data Analysis and Reporting System (CDARS) database. The CDARS was developed and managed by the Hong Kong Hospital Authority, which provides public healthcare services, including primary, secondary, and tertiary care, to more than seven million Hong Kong residents. This database contains patient‐level records, including demographic details and clinical information on diagnoses, operations, prescriptions, visits to accident and emergency departments, hospitals, and outpatient clinics. To protect patient confidentiality, anonymous patient identifiers were generated to allow the linkage of all clinical information. This database has been used to conduct high‐quality epidemiological studies [39, 40, 41, 42, 43].

### 2.3 Patient Identification

Patients who had a diagnostic record of ischaemic stroke as the principal diagnosis in an inpatient setting or an accident and emergency department attendance were included during the study period (January 1, 2003, to December 31, 2014). Only their incident event was selected as the outcome of interest for the analysis. The diagnosis was identified using the International Classification of Diseases, Ninth Revision, Clinical Modification (ICD‐9‐CM) code (Supporting Information 2). Previous research has demonstrated a high positive predictive value (90%) for ischemic stroke diagnosis in the CDARS [44].

The exposure of interest is the oral PPI prescriptions prescribed in the outpatient clinics. The British National Formulary Chapter was used to identify the drug use history. (Supporting Information 1) We excluded patients who received any PPI prescription 3 years before the study start date to identify incident PPI users. Patients with non‐oral PPI or PPIs prescribed during hospital stay before the first exposure of interest were also excluded.

Unlike a standard SCCS that included individuals with both events and exposure, the modified SCCS also included those ischemic stroke individuals without PPI use history during the observation period [33].

### 2.4 Observation and Risk Periods Definition in SCCS Models

**FIGURE 1:**
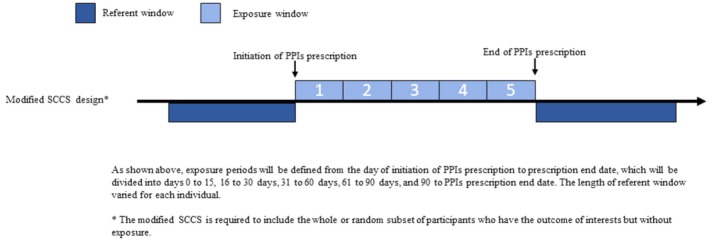
Illustration of the modified self‐controlled case series designs.

The observation period began on January 1, 2003, and concluded on December 31, 2014. The exposure window started from the prescription start to the end date of PPIs, subdivided into Days 0–30, 31–60, 61–90, and 91 to the end of the prescription. Continuous exposure to PPIs was assumed when there is a treatment gap of ≤ 30 days between consecutive treatments to account for any potential medication stockpiling and non‐adherence. All other non‐exposure windows were defined as referent windows. The study schemas for study design are illustrated in Figure 1.

### 2.5 Statistical Analysis

We estimated incidence rate ratios (IRRs) by comparing the incidence of ischaemic stroke hospital admission among exposure and referent windows. The estimation process in the modified SCCS relies on estimating equations with a pseudo‐Poisson model [33, 35], deviating from the Poisson‐likelihood method used in the standard SCCS [34, 35]. The process was carried out by reweighting observations in a way that they conform to the counterfactual that all exposures after an event are disregarded, and maximizing a Poisson pseudo‐likelihood. It can then produce the correct estimating equations to obtain the IRRs [33, 35]. We further adjusted for the age effect using a one‐year band. All analyses were performed in R version 4.1.2 (R Foundation for Statistical Computing, Vienna, Austria) with the R‐package “SCCS” version 1.6.

### 2.6 Stratified and Sensitivity Analysis

Further, as antithrombotic drugs, including anticoagulants and antiplatelets, might be prescribed with PPIs for the primary prevention of stroke in patients with cardiovascular disease, the degree of the SCCS assumption violation could be different in people who had concomitant use of antithrombotic drugs and PPIs from those who had PPI monotherapy. Patients were categorized as co‐prescription users when having any overlapping prescription records of PPI and antithrombotic drugs. We, therefore, stratified the analysis according to the co‐prescribed antithrombotic drugs in the modified SCCS.

We conducted sensitivity analyses to test the robustness of the primary analysis. We assessed the robustness of the definition of exposure windows by (1) sub‐dividing exposure windows using other intervals: Days 0–60, 61–120, 121 to prescription end date to assess whether the choice of exposure window length influenced the estimation results; (2) collapsing the exposure window into a single period spanning from the start of the prescription to the end of the prescription to obtain the IRR for an overall prescription period.

## 3 Results

**FIGURE 2:**
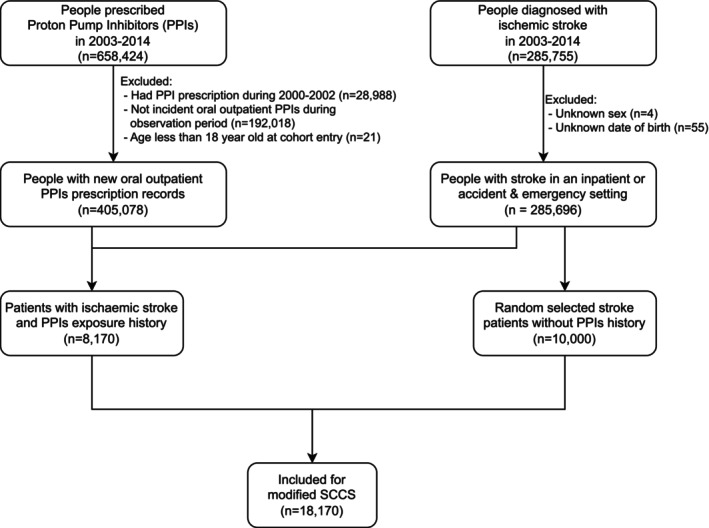
Flowchart of participant inclusion and exclusion in the self‐controlled case series.

Figure 2 shows the flow of participants' inclusion. During the study period, we identified 658 424 individuals with at least one PPI prescription in the outpatient clinics. Within this cohort, 8170 patients had both PPI prescriptions and experienced an incident of ischaemic stroke during the observation period. The median prescription period for PPI was 55 days, with a fixed observation period of 12.0 years for all participants. Pantoprazole and esomeprazole were the most prescribed PPIs among the identified patients (Supporting Information 3). Their average age at cohort entry was 71.1 (standard deviation: 13.7) years; male participants accounted for 52% of the study cohort. Supporting Information 4 shows the characteristics of PPI users on the prescription start date. An additional 10 000 participants who had an ischaemic stroke episode but did not receive PPI prescriptions were selected at random and included in the analysis for modified SCCS.

**FIGURE 3:**
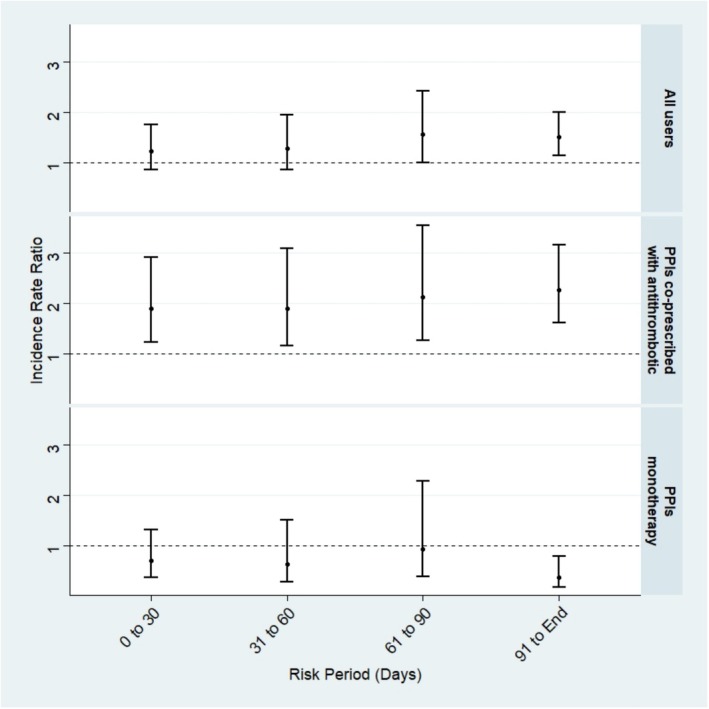
Incidence rate ratio of ischaemic stroke among PPI users, stratified by antithrombotic drug users.

Figure 3 and Table 1 present the IRRs from the modified SCCS of primary and stratified analyses. In the primary analysis including all PPI users, there was no increased risk of ischaemic stroke, except a marginal increase from Days 61 to 91 after the prescription start date (IRR: 1.55; 95% CI: 1.00–2.42) and an increased risk of ischaemic stroke during Days 90 after prescription start date of PPIs to prescription end date (IRR: 1.51; 95% CI: 1.14–2.00) (Table 1). In the subgroup analysis (Table 2), 5595 patients were prescribed antithrombotic drugs concurrently with PPIs during the observation period. By limiting the analysis among these co‐prescribed antithrombotic drugs users, there was an increased risk of stroke in all risk versus referent windows. Among people only with PPI monotherapy (*N* = 2575), we did not observe a higher risk of ischaemic stroke with wide CIs but a notably lower risk for the exposure window of 90 days after PPI prescription (IRR: 0.37 [95% CI 0.18–0.78]). All sensitivity analyses showed similar results to the primary analysis (Table 1).

**TABLE 1:** Incidence rate ratios for ischaemic stroke following the use of oral outpatient proton pump inhibitor prescriptions using modified SCCS.

	Total follow‐up (years)	Number of events	Incidence rate ratio (95% CI)
Primary analysis			
Referent window	212 721.69	11 617	—
Days 0–30	610.24	37	1.22 (0.86–1.74)
Days 31–60	442.67	24	1.28 (0.85–1.94)
Days 61–90	363.87	21	1.55 (1.00–2.42)
Days 91‐End	3901.53	112	1.51 (1.14–2.00)
Sensitivity analysis—Different pre‐defined risk windows			
Referent window	212 721.69	11 617	—
Days 0–60	1052.91	61	1.24 (0.94–1.65)
Days 61–120	671.71	35	1.48 (1.04–2.10)
Days 121‐End	3593.69	98	1.54 (1.14–2.09)
Sensitivity analysis—Combining exposure windows			
Referent window	254 002.57	11 617	—
Days 0‐End	5989.43	194	1.40 (1.14–1.71)

**TABLE 2:** Risk of ischaemic stroke associated with proton pump inhibitors stratified by antithrombotic drug users using modified SCCS.

	PPI co‐prescribed antithrombotic drugs users			PPI monotherapy users		
	Total follow‐up (years)	Number of events	Incidence rate ratio (95% CI)	Total follow‐up (years)	Number of events	Incidence rate ratio (95% CI)
Referent window	182 468.61	9657	—	150 253.08	7672	
Days 0–30	451.58	27	1.89 (1.23–2.91)	158.66	10	0.69 (0.37–1.31)
Days 31–60	353.57	19	1.90 (1.17–3.08)	29.09	5	0.63 (0.27–1.51)
Days 61–90	301.51	16	2.12 (1.26–3.55)	62.35	5	0.93 (0.38–2.27)
Days 91–end	3564.72	104	2.26 (1.61–3.16)	336.81	8	0.37 (0.18–0.78)

Abbreviation: PPIs, proton pump inhibitors.

## 4 Discussion

This is the first study adopting modified SCCS investigating drug safety outside the context of COVID‐19 vaccine research, with adequate adjustments when assumptions are violated. Our study shows a higher risk of ischaemic stroke from Days 61 to 90 and from Days 91 to the prescription end for all PPI users, but not in other risk windows versus referent windows. The association may be modified by using antithrombotic drugs, which is understudied in current literature. In those with PPIs co‐prescribed with antithrombotic drugs, the risk of ischaemic stroke was higher in all exposure windows after initiating a PPI prescription compared with the referent window. However, in people with PPI monotherapy, no evidence of a higher risk of ischaemic stroke during all risk versus referent windows was found. We also identified a lower risk of ischaemic stroke from Day 91 to the end of prescription versus referent windows.

The association between PPIs and the risk of incident ischemic stroke remains a topic of debate and the evidence is conflicting [18, 45, 46]. First, previous studies reported that an increased risk of ischemic stroke after PPI use may be influenced by residual confounding. A Taiwanese observational study reported a 36% increase in the risk of stroke associated with PPIs within 120 days using a cohort design (hazard ratio [HR]: 1.36, 1.14–1.62) [18]. In their nested case–control study design, the highest odds ratio was within 30 days (OR: 1.77; 95% CI: 1.45–2.18), gradually decreased between 31 and 90 days (OR: 1.65; 95% CI: 1.31–2.08) and 90 and 180 days (OR: 1.28; 95% CI: 1.03–1.59) [18]. Both designs are susceptible to confounding due to the risk comparison between people with systematic health differences. In contrast, the SCCS design has the advantage of inherent control for time‐invariant confounders. Our modified SCCS analysis did not detect higher ischemic stroke risk in patients with PPI monotherapy. Further, of 658 424 individuals who were ever prescribed a PPI, we only identified a very low number of people (*n* = 2633) receiving a PPI monotherapy who also subsequently had an ischemic stroke. It demonstrated that the absolute risk of ischemic stroke was minimal in people prescribed PPI monotherapy.

Secondly, the higher risk of ischaemic stroke observed in previous studies could be partly explained by the higher baseline risk of ischaemic stroke. Guidelines for antithrombotic drug therapy in the primary prevention of ischaemic stroke recommend that the risk of bleeding should be considered and suggest PPI prescription when necessary [26, 27]. Therefore, PPI users could be commonly co‐prescribed with antithrombotic drugs and are at high risk of developing ischaemic stroke. Our stratified modified SCCS found a higher risk of ischaemic stroke among PPI users co‐prescribed with antithrombotic drug users rather than in those receiving PPI monotherapy. Previous studies also suggested that antithrombotic drug and PPI co‐prescribed users have systematic differences in their baseline characteristics compared to non‐PPI users, which could lead to spurious associations [47, 48]. A cohort study using data from the Nurses' Health Study found that the risk of ischemic stroke after prescribing PPIs substantially attenuated after confounding adjustment, including past use of aspirin [46]. The association reported from the UK Biobank database was weakened (HR: 1.14, 1.04–1.26 vs. 1.48, 1.37–1.61) after including Histamine‐2 Receptor Antagonist (H2RA) and antithrombotic drugs as confounders in the multivariable models [10]. A comprehensive PPI safety study on 15 outcomes was conducted using UK, USA, and Chinese databases [28]. Although a higher risk of ischaemic stroke was identified after PPI use (HR: 1.13, 1.06–1.21), they only adjusted for aspirin use but not for other antithrombotic drugs. In a double‐blind randomized clinical trial, no difference in the risk of stroke was also found following pantoprazole, aligning with our results in the stratified analysis only containing PPI monotherapy [49]. The relatively smaller risk in our modified SCCS including all PPI users, compared with antithrombotic drug co‐prescribed users, could be due to the mixed cohort with a null effect from PPI monotherapy users. Previous studies also suggested that the relationship between PPIs and stroke may be influenced by the presence of antithrombotic drug medications, but the role of antithrombotic drugs in such association was still understudied [50]. Given that there is a proposed mechanism that PPIs could interact with metabolic enzymes like CYP2C19, which could potentially reduce the cardiovascular benefit from antithrombotic [50, 51], further analyses need to be conducted to explore the role of antithrombotic drugs in the risk of ischaemic stroke among PPI users. Besides, a time‐dependent pattern was observed in the overall population, with increased risk occurring after 60 days, and among co‐prescribed antithrombotic users, where risk estimates were increasing during the prescription period. These findings align with existing literature indicating that PPI‐associated risks are predominantly linked to long‐term use rather than short‐term usage [5, 45, 52]. Further research is necessary to confirm this association.

In addition, our finding was consistent with an elderly case–control study in Israel, where a protective effect of PPIs was identified after excluding participants with a history of antiplatelet use [9]. The protective effect in their study was more pronounced among long‐term PPI users (HR: 0.51; 95% CI: 0.46–0.55), while in our analysis, the protection among PPI monotherapy was specifically observed from 91 days after PPI prescription (IRR for Days 91‐End: 0.37; [95% CI: 0.18–0.78]). However, due to the small sample size for people with PPI monotherapy in our current study, further investigation is required.

Assumptions assessment is crucial in a SCCS analysis. A recent study in Korea using a standard SCCS identified no increased risk of ischaemic stroke after the prescription of PPIs [53]. Notably, we showed that the standard SCCS violated the assumptions of both event‐dependent exposure and event‐dependent observation. Despite adding a pre‐exposure window can potentially address the event‐dependent exposure as a conventional approach, it offers no solutions when another assumption is violated when there is a higher risk of mortality shortly after occurring ischaemic stroke. Therefore, standard SCCS might not be appropriate in this research question. The modified SCCS has been developed to address both assumptions violations that have been widely applied in the COVID‐19 vaccine safety studies [30, 33, 36]. The current analysis is the first study applying modified SCCS outside COVID‐19 vaccines studies.

This is the first population‐based study investigating the association between ischaemic stroke and the use of PPIs with routine clinical data from over 658 000 individuals using the modified SCCS. We also carefully assessed the assumption of SCCS and performed a stratified analysis to explore the association. However, this study is subject to a few limitations. First, similar to most databases, there is no data on drug compliance in the CDARS. This could result in misclassification bias of exposure status. However, we assumed continuous exposure of drug exposure when there is a treatment gap of less than or equal to 30 days to reduce the misclassification bias. Second, the CDARS does not capture the clinical data from the private healthcare sector. However, our study included patients who visited the public healthcare sector at least twice during the observation period, inferring that they utilize healthcare services mainly in the public sector given the low consultation and drug costs. Thirdly, it is possible to have potential time‐varying confounders in the SCCS study design. To partially address this issue, we attempted to incorporate the effect of age into the model for adjustment. Fourthly, given that the data used in this study ended in 2014, the prescribing pattern and cardiovascular care may have changed after 2014. However, the change in prescribing pattern and cardiovascular care should not affect the performance of the self‐controlled case series design. Lastly, while the current analysis focused on ischemic stroke, future studies with a larger sample size could investigate the risk of haemorrhagic stroke associated with PPIs to further understand their potential pathways of thrombotic and bleeding risks.

## 5 Conclusion

We demonstrated an increased risk of ischaemic stroke in people co‐prescribed antithrombotic drugs and PPIs, but not in those in PPI monotherapy. The higher risk of ischaemic stroke observed in people co‐prescribed antithrombotic drugs and PPIs may be due to their underlying higher risk profiles. Modified SCCS should be considered when both assumptions of event‐dependent exposure and event‐dependent observation periods are likely to be violated.

### 5.1 Plain Language Summary

Previous studies suggest that common stomach acid medications (PPIs) might be linked to stroke, but results have been unclear. This study explored whether such a link could be due to other factors, such as people's existing health risks. For instance, some patients take PPIs to protect their stomachs while using blood‐thinning medications (like aspirin), which themselves might affect stroke risk. We analyzed 18 170 adults in Hong Kong who had a stroke between 2003 and 2014. We compared stroke risk during time periods after starting PPIs to times when patients were not using these PPIs. Overall, there was a small increase in stroke risk 2–3 months after starting PPIs. However, when focusing on the patients taking PPIs alone (not with blood thinners), it showed no increased risk. The higher risk was only seen in people prescribed both PPIs and blood thinners.

## Author Contributions

C.S.L.C., A.Y.S.W., E.W.Y.C., and I.C.K.W. had the original idea for this study. C.S.L.C., A.Y.S.W., and M.F. contribute to the development of the study design. A.Y.S.W., C.S.L.C., J.E.B., and J.Z. extracted data from the source database. M.F., A.Y.S.W., and J.E.B. undertook the analyses. M.F., A.Y.S.W., and C.S.L.C. wrote the first draft of the paper. M.F., J.E.B., I.C.K.W., J.Z., K.S.C., E.W.Y.C., A.Y.S.W., and C.S.L.C. provided critical input to the discussion. All authors contributed to the interpretation of the analyses, reviewing and editing of the manuscript, as well as approved the final manuscript.

## Disclosure

I.C.K.W. reports grants from Amgen, Bristol‐Myers Squibb, Pfizer, Janssen, Bayer, GSK, and Novartis, the Hong Kong Researches Grant Council, and the Hong Kong Health and Medical Research Fund in Hong Kong; National Institute for Health Research in England; European Commission; National Health and Medical Research Council in Australia; consulting fees from IQVIA and World Health Organization; payment for expert testimony for the Appeal Court of Hong Kong; and is a non‐executive director of Jacobson Medical in Hong Kong and Therakind in England, outside of the submitted work; no other relationships or activities that could appear to have influenced the submitted work. E.W.Y.C. reports grants from the Research Grants Council (RGC, Hong Kong), Research Fund Secretariat of the Food and Health Bureau, National Natural Science Fund of China, Wellcome Trust, Bayer, Bristol‐Myers Squibb, Pfizer, Janssen, Amgen, Takeda, and Narcotics Division of the Security Bureau of HKSAR, and an honorarium from the Hospital Authority, outside the submitted work. C.S.L.C. has received grants from the Food and Health Bureau of the Hong Kong Government, Hong Kong Research Grant Council, Hong Kong Innovation and Technology Commission, Pfizer, IQVIA, and Amgen, and personal fees from Primevigilance, outside the submitted work. A.Y.S.W. holds a fellowship (FS/19/19/34175) from British Heart Foundation. Funders had no role in the study design, collection, analysis, and interpretation of data; in the writing of the report; and in the decision to submit the article for publication.

## Conflicts of Interest

The authors declare no conflicts of interest.

## Supporting information


**Data S1:** pds70219‐sup‐0001‐supinfo.docx.

## Data Availability

Data are available on reasonable request. Data from the study can be requested from the corresponding author.

## References

[1] NICE , “Gastro‐Oesophageal Reflux Disease and Dyspepsia in Adults: Investigation and Management,” (2014), http://www.nice.org.uk/guidance/cg184/resources/gastrooesophageal‐reflux‐disease‐and‐dyspepsia‐in‐adults‐investigation‐and‐management‐35109812699845. 31935049

[2] P. Malfertheiner , F. Megraud , C. A. O'Morain , et al., “Management of *Helicobacter Pylori* Infection‐The Maastricht V/Florence Consensus Report,” Gut 66, no. 1 (2017): 6–30. 27707777 10.1136/gutjnl-2016-312288

[3] W. Gomm , K. von Holt , F. Thome , et al., “Association of Proton Pump Inhibitors With Risk of Dementia: A Pharmacoepidemiological Claims Data Analysis,” JAMA Neurology 73, no. 4 (2016): 410–416. 26882076 10.1001/jamaneurol.2015.4791

[4] C. Caetano , M. Veloso , and S. Borda , “Proton Pump Inhibitors and Dementia: What Association?,” Dementia & Neuropsychologia 17 (2023): e20220048. 37261259 10.1590/1980-5764-DN-2022-0048 PMC10229084

[5] K. S. Cheung , E. W. Chan , A. Y. S. Wong , L. Chen , I. C. K. Wong , and W. K. Leung , “Long‐Term Proton Pump Inhibitors and Risk of Gastric Cancer Development After Treatment for *Helicobacter pylori*: A Population‐Based Study,” Gut 67, no. 1 (2018): 28–35. 29089382 10.1136/gutjnl-2017-314605

[6] D. N. Juurlink , C. R. Dormuth , A. Huang , et al., “Proton Pump Inhibitors and the Risk of Adverse Cardiac Events,” PLoS One 8, no. 12 (2013): e84890. 24386430 10.1371/journal.pone.0084890 PMC3873988

[7] N. H. Shah , P. LePendu , A. Bauer‐Mehren , et al., “Proton Pump Inhibitor Usage and the Risk of Myocardial Infarction in the General Population,” PLoS One 10, no. 6 (2015): e0124653. 26061035 10.1371/journal.pone.0124653 PMC4462578

[8] C. J. Shih , Y. T. Chen , S. M. Ou , S. Y. Li , T. J. Chen , and S. J. Wang , “Proton Pump Inhibitor Use Represents an Independent Risk Factor for Myocardial Infarction,” International Journal of Cardiology 177, no. 1 (2014): 292–297. 25499395 10.1016/j.ijcard.2014.09.036

[9] H. Schmilovitz‐Weiss , R. Gingold‐Belfer , N. Peleg , et al., “Use of Proton Pump Inhibitors Is Associated With Lower Rates of First‐Time Ischemic Stroke in Community‐Dwelling Elderly,” British Journal of Clinical Pharmacology 87, no. 3 (2021): 1187–1193. 32692459 10.1111/bcp.14488

[10] M. Yang , Q. He , F. Gao , et al., “Regular Use of Proton‐Pump Inhibitors and Risk of Stroke: A Population‐Based Cohort Study and Meta‐Analysis of Randomized‐Controlled Trials,” BMC Medicine 19, no. 1 (2021): 316. 34856983 10.1186/s12916-021-02180-5 PMC8641218

[11] S. Abedini , A. Meinitzer , I. Holme , et al., “Asymmetrical Dimethylarginine Is Associated With Renal and Cardiovascular Outcomes and All‐Cause Mortality in Renal Transplant Recipients,” Kidney International 77, no. 1 (2010): 44–50. 19847152 10.1038/ki.2009.382

[12] R. H. Boger , H. G. Endres , E. Schwedhelm , et al., “Asymmetric Dimethylarginine as an Independent Risk Marker for Mortality in Ambulatory Patients With Peripheral Arterial Disease,” Journal of Internal Medicine 269, no. 3 (2011): 349–361. 21175900 10.1111/j.1365-2796.2010.02322.x

[13] S. Kiechl , T. Lee , P. Santer , et al., “Asymmetric and Symmetric Dimethylarginines Are of Similar Predictive Value for Cardiovascular Risk in the General Population,” Atherosclerosis 205, no. 1 (2009): 261–265. 19100547 10.1016/j.atherosclerosis.2008.10.040

[14] T. Leong , D. Zylberstein , I. Graham , et al., “Asymmetric Dimethylarginine Independently Predicts Fatal and Nonfatal Myocardial Infarction and Stroke in Women: 24‐Year Follow‐Up of the Population Study of Women in Gothenburg,” Arteriosclerosis, Thrombosis, and Vascular Biology 28, no. 5 (2008): 961–967. 18292394 10.1161/ATVBAHA.107.156596

[15] R. Schnabel , S. Blankenberg , E. Lubos , et al., “Asymmetric Dimethylarginine and the Risk of Cardiovascular Events and Death in Patients With Coronary Artery Disease: Results From the AtheroGene Study,” Circulation Research 97, no. 5 (2005): e53–e59. 16100045 10.1161/01.RES.0000181286.44222.61

[16] G. Tripepi , F. Mattace‐Raso , F. Rapisarda , et al., “Traditional and Nontraditional Risk Factors as Predictors of Cerebrovascular Events in Patients With End Stage Renal Disease,” Journal of Hypertension 28, no. 12 (2010): 2468–2474. 20724936 10.1097/HJH.0b013e32833eaf49

[17] C. Zoccali , S. Bode‐Boger , F. Mallamaci , et al., “Plasma Concentration of Asymmetrical Dimethylarginine and Mortality in Patients With End‐Stage Renal Disease: A Prospective Study,” Lancet 358, no. 9299 (2001): 2113–2117. 11784625 10.1016/s0140-6736(01)07217-8

[18] Y. F. Wang , Y. T. Chen , J. C. Luo , T. J. Chen , J. C. Wu , and S. J. Wang , “Proton‐Pump Inhibitor Use and the Risk of First‐Time Ischemic Stroke in the General Population: A Nationwide Population‐Based Study,” American Journal of Gastroenterology 112, no. 7 (2017): 1084–1093. 28397874 10.1038/ajg.2017.101

[19] P. Willeit , D. F. Freitag , J. A. Laukkanen , et al., “Asymmetric Dimethylarginine and Cardiovascular Risk: Systematic Review and Meta‐Analysis of 22 Prospective Studies,” Journal of the American Heart Association 4, no. 6 (2015): e001833. 26021436 10.1161/JAHA.115.001833 PMC4599532

[20] Y. T. Ghebremariam , J. P. Cooke , F. Khan , et al., “Proton Pump Inhibitors and Vascular Function: A Prospective Cross‐Over Pilot Study,” Vascular Medicine 20, no. 4 (2015): 309–316. 25835348 10.1177/1358863X14568444 PMC4572842

[21] A. A. Root , A. Y. Wong , Y. Ghebremichael‐Weldeselassie , et al., “Evaluation of the Risk of Cardiovascular Events With Clarithromycin Using Both Propensity Score and Self‐Controlled Study Designs,” British Journal of Clinical Pharmacology 82, no. 2 (2016): 512–521. 27090996 10.1111/bcp.12983 PMC4972168

[22] A. Ormeci , Z. Emrence , B. Baran , et al., “Can *Helicobacter pylori* Be Eradicated With High‐Dose Proton Pump Inhibitor in Extensive Metabolizers With the CYP2C19 Genotypic Polymorphism?,” European Review for Medical and Pharmacological Sciences 20, no. 9 (2016): 1795–1797. 27212172

[23] Y. Wu , Z. Tao , C. Song , et al., “Overexpression of YKL‐40 Predicts Plaque Instability in Carotid Atherosclerosis With CagA‐Positive *Helicobacter pylori* Infection,” PLoS One 8, no. 4 (2013): e59996. 23573226 10.1371/journal.pone.0059996 PMC3616092

[24] A. Y. Wong , A. Root , I. J. Douglas , et al., “Cardiovascular Outcomes Associated With Use of Clarithromycin: Population Based Study,” BMJ (Clinical Research Ed) 352 (2016): h6926. 10.1136/bmj.h6926 26768836

[25] C. S. L. Chui , K. S. Cheung , J. P. Brown , et al., “Proton Pump Inhibitors and Myocardial Infarction: An Application of Active Comparators in a Self‐Controlled Case Series,” International Journal of Epidemiology 52, no. 3 (2023): 899–907. 36259933 10.1093/ije/dyac196 PMC10244046

[26] A. Greco , G. Occhipinti , D. Giacoppo , et al., “Antithrombotic Therapy for Primary and Secondary Prevention of Ischemic Stroke: JACC State‐of‐the‐Art Review,” Journal of the American College of Cardiology 82, no. 15 (2023): 1538–1557. 37793752 10.1016/j.jacc.2023.07.025

[27] D. J. Kumbhani , C. P. Cannon , C. J. Beavers , et al., “2020 ACC Expert Consensus Decision Pathway for Anticoagulant and Antiplatelet Therapy in Patients With Atrial Fibrillation or Venous Thromboembolism Undergoing Percutaneous Coronary Intervention or With Atherosclerotic Cardiovascular Disease: A Report of the American College of Cardiology Solution Set Oversight Committee,” Journal of the American College of Cardiology 77, no. 5 (2021): 629–658. 33250267 10.1016/j.jacc.2020.09.011

[28] B. Xia , Q. He , F. G. Smith , et al., “Individualized Prevention of Proton Pump Inhibitor Related Adverse Events by Risk Stratification,” Nature Communications 15, no. 1 (2024): 3591. 10.1038/s41467-024-48007-8 PMC11055952 38678022

[29] J. P. Brown , J. R. Tazare , E. Williamson , et al., “Proton Pump Inhibitors and Risk of All‐Cause and Cause‐Specific Mortality: A Cohort Study,” British Journal of Clinical Pharmacology 87, no. 8 (2021): 3150–3161. 33393677 10.1111/bcp.14728 PMC11497312

[30] C. S. L. Chui , M. Fan , E. Y. F. Wan , et al., “Thromboembolic Events and Hemorrhagic Stroke After mRNA (BNT162b2) and Inactivated (CoronaVac) Covid‐19 Vaccination: A Self‐Controlled Case Series Study,” EClinicalMedicine 50 (2022): 101504. 35770253 10.1016/j.eclinm.2022.101504 PMC9233170

[31] M. Patone , X. W. Mei , L. Handunnetthi , et al., “Risks of Myocarditis, Pericarditis, and Cardiac Arrhythmias Associated With COVID‐19 Vaccination or SARS‐CoV‐2 Infection,” Nature Medicine 28, no. 2 (2022): 410–422. 10.1038/s41591-021-01630-0 PMC8863574 34907393

[32] J. Hippisley‐Cox , M. Patone , X. W. Mei , et al., “Risk of Thrombocytopenia and Thromboembolism After Covid‐19 Vaccination and SARS‐CoV‐2 Positive Testing: Self‐Controlled Case Series Study,” BMJ 374 (2021): n1931. 34446426 10.1136/bmj.n1931 PMC8388189

[33] Y. Ghebremichael‐Weldeselassie , M. J. Jabagi , J. Botton , et al., “A Modified Self‐Controlled Case Series Method for Event‐Dependent Exposures and High Event‐Related Mortality, With Application to COVID‐19 Vaccine Safety,” Statistics in Medicine 41, no. 10 (2022): 1735–1750. 35092037 10.1002/sim.9325 PMC9303905

[34] H. J. Whitaker , Y. Ghebremichael‐Weldeselassie , I. J. Douglas , L. Smeeth , and C. P. Farrington , “Investigating the Assumptions of the Self‐Controlled Case Series Method,” Statistics in Medicine 37, no. 4 (2018): 643–658. 29094391 10.1002/sim.7536

[35] P. Farrington , H. Whitaker , and Y. Ghebremichael‐Weldeselassie , Self‐Controlled Case Series Studies: A Modelling Guide With R, 1st ed. (Chapman and Hall/CRC, 2018).

[36] M. Fan , F. T. T. Lai , F. W. T. Cheng , et al., “Risk of Carditis After Three Doses of Vaccination With mRNA (BNT162b2) or Inactivated (CoronaVac) Covid‐19 Vaccination: A Self‐Controlled Cases Series and a Case‐Control Study,” Lancet Regional Health 35 (2023): 100745. 10.1016/j.lanwpc.2023.100745 PMC10039395 37360861

[37] C. W. Sing , C. T. L. Tang , C. S. L. Chui , et al., “COVID‐19 Vaccines and Risks of Hematological Abnormalities: Nested Case‐Control and Self‐Controlled Case Series Study,” American Journal of Hematology 97, no. 4 (2022): 470–480. 35080247 10.1002/ajh.26478 PMC9011752

[38] E. Y. F. Wan , C. S. L. Chui , Y. Wang , et al., “Herpes Zoster Related Hospitalization After Inactivated (CoronaVac) and mRNA (BNT162b2) SARS‐CoV‐2 Vaccination: A Self‐Controlled Case Series and Nested Case‐Control Study,” Lancet Regional Health 21 (2022): 21. 10.1016/j.lanwpc.2022.100393 PMC8808060 35128500

[39] E. W. Chan , W. C. Lau , W. K. Leung , et al., “Prevention of Dabigatran‐Related Gastrointestinal Bleeding With Gastroprotective Agents: A Population‐Based Study,” Gastroenterology 149, no. 3 (2015): 586–595.e3. 25960019 10.1053/j.gastro.2015.05.002

[40] C. S. Chui , E. W. Chan , A. Y. Wong , A. Root , I. J. Douglas , and I. C. Wong , “Association Between Oral Fluoroquinolones and Seizures: A Self‐Controlled Case Series Study,” Neurology 86, no. 18 (2016): 1708–1715. 27053716 10.1212/WNL.0000000000002633 PMC4854590

[41] W. C. Lau , E. W. Chan , C. L. Cheung , et al., “Association Between Dabigatran vs Warfarin and Risk of Osteoporotic Fractures Among Patients With Nonvalvular Atrial Fibrillation,” JAMA 317, no. 11 (2017): 1151–1158. 28324091 10.1001/jama.2017.1363

[42] K. K. C. Man , E. W. Chan , P. Ip , et al., “Prenatal Antidepressant Use and Risk of Attention‐Deficit/Hyperactivity Disorder in Offspring: Population Based Cohort Study,” BMJ 357 (2017): j2350. 28566274 10.1136/bmj.j2350 PMC5450015

[43] A. Y. Wong , I. C. Wong , C. S. Chui , et al., “Association Between Acute Neuropsychiatric Events and *Helicobacter pylori* Therapy Containing Clarithromycin,” JAMA Internal Medicine 176, no. 6 (2016): 828–834. 27136661 10.1001/jamainternmed.2016.1586

[44] E. W. Chan , W. C. Lau , C. W. Siu , et al., “Effect of Suboptimal Anticoagulation Treatment With Antiplatelet Therapy and Warfarin on Clinical Outcomes in Patients With Nonvalvular Atrial Fibrillation: A Population‐Wide Cohort Study,” Heart Rhythm 13, no. 8 (2016): 1581–1588. 27033342 10.1016/j.hrthm.2016.03.049

[45] T. S. G. Sehested , T. A. Gerds , E. L. Fosbol , et al., “Long‐Term Use of Proton Pump Inhibitors, Dose‐Response Relationship and Associated Risk of Ischemic Stroke and Myocardial Infarction,” Journal of Internal Medicine 283, no. 3 (2018): 268–281. 29024109 10.1111/joim.12698

[46] L. H. Nguyen , P. Lochhead , A. D. Joshi , et al., “No Significant Association Between Proton Pump Inhibitor Use and Risk of Stroke After Adjustment for Lifestyle Factors and Indication,” Gastroenterology 154, no. 5 (2018): 1290–1297.e1. 29269313 10.1053/j.gastro.2017.12.006 PMC5880683

[47] R. N. Cardoso , A. M. Benjo , J. J. DiNicolantonio , et al., “Incidence of Cardiovascular Events and Gastrointestinal Bleeding in Patients Receiving Clopidogrel With and Without Proton Pump Inhibitors: An Updated Meta‐Analysis,” Open Heart 2, no. 1 (2015): e000248. 26196021 10.1136/openhrt-2015-000248 PMC4488889

[48] C. Melloni , J. B. Washam , W. S. Jones , et al., “Conflicting Results Between Randomized Trials and Observational Studies on the Impact of Proton Pump Inhibitors on Cardiovascular Events When Coadministered With Dual Antiplatelet Therapy: Systematic Review,” Circulation. Cardiovascular Quality and Outcomes 8, no. 1 (2015): 47–55. 25587094 10.1161/CIRCOUTCOMES.114.001177 PMC6143138

[49] P. Moayyedi , J. W. Eikelboom , J. Bosch , et al., “Safety of Proton Pump Inhibitors Based on a Large, Multi‐Year, Randomized Trial of Patients Receiving Rivaroxaban or Aspirin,” Gastroenterology 157, no. 3 (2019): 682–691.e2. 31152740 10.1053/j.gastro.2019.05.056

[50] K. Malhotra , A. H. Katsanos , M. Bilal , M. F. Ishfaq , N. Goyal , and G. Tsivgoulis , “Cerebrovascular Outcomes With Proton Pump Inhibitors and Thienopyridines: A Systematic Review and Meta‐Analysis,” Stroke 49, no. 2 (2018): 312–318. 29339434 10.1161/STROKEAHA.117.019166

[51] S. Agewall , M. Cattaneo , J. P. Collet , et al., “Expert Position Paper on the Use of Proton Pump Inhibitors in Patients With Cardiovascular Disease and Antithrombotic Therapy,” European Heart Journal 34, no. 23 (2013): 1708–1713. 23425521 10.1093/eurheartj/eht042

[52] D. E. Freedberg , L. S. Kim , and Y. X. Yang , “The Risks and Benefits of Long‐Term Use of Proton Pump Inhibitors: Expert Review and Best Practice Advice From the American Gastroenterological Association,” Gastroenterology 152, no. 4 (2017): 706–715. 28257716 10.1053/j.gastro.2017.01.031

[53] J. Y. Park , J. Yoo , J. Jeon , J. Kim , and S. Kang , “Proton Pump Inhibitors and Risk of Cardiovascular Disease: A Self‐Controlled Case Series Study,” American Journal of Gastroenterology 117, no. 7 (2022): 1063–1071. 35505518 10.14309/ajg.0000000000001809

