# Supplementary material for: Risk of Ischaemic Stroke Varies With Antithrombotic Drugs Use in Proton Pump Inhibitor Users: A Self‐Controlled Case Series Study
Source: Pharmacoepidemiol Drug Saf. 2025 Sep 18;34(9):e70219. doi: 10.1002/pds.70219 (PMC12445257; doi:10.1002/pds.70219)
Supplement: Supplementary file 1 — Data S1: pds70219‐sup‐0001‐supinfo.docx. [file PDS-34-e70219-s001.docx]

# Supplementary materials

## Supplementary 1. Assumptions for standard SCCS

There are three major SCCS assumptions(1, 2) to be fulfilled for unbiased estimates in the standard SCCS method: 1) the occurrence of events should be independent; 2) events should not temporarily alter the occurrence of subsequent exposure (event-dependent exposure); and 3) events should not alter the end of the observation period (event-dependent observation).(1-3)

When there is a violation of assumption 1), the analyses can focus on only their first recorded events. Diagnostic analyses can be performed by plotting two histograms to assess the other violations of assumptions 2) and 3).(2, 3)

The first histogram examined the assumption of event-dependent exposure by plotting the frequency of the cases against the interval between exposures and events by day, with the date of events occurrence defined as Day 0. If there is a pattern of an abrupt mode, increase or decrease, shortly before Day 0, it indicates a high possibility of event-dependent exposure issues. A common approach to deal with event-dependent exposure is introducing a period shortly before the predefined exposure window (pre-exposure window) with a pre-specified duration. Therefore, the pre-exposure window can be removed from the referent window to avoid the distortion of the rates during the referent window. However, the length of the pre-exposure window is usually arbitrarily predefined, and the performance of introducing the pre-exposure window to address this assumption has also been understudied.

The second histogram examined the assumption of event-dependent observation by plotting the frequency of the cases against the interval between the events and the end of the observation period, stratified by whether the observation period was censored (i.e. censored at death date) or not (i.e. without any censoring even for death patients). If there is an abrupt mode in the interval representing the time shortly after the events in the censored figure compared with the uncensored figure, it would highly suggest an event-dependent observation issue. When the number of event-related mortality is low, the common approach to deal with event-dependent observation is to conduct sensitivity analysis by excluding people who died in a short period after outcome occurrence to compare the robustness of the findings. (1, 2) However, it could reduce precision to detect meaningful estimation.

The following figure 1 illustrates the assumption checking for event-dependent issues in this study, stratified by antithrombotic users as well. Panel A shown the assumption check for event-dependent exposure issue. We observed a sharp increase in case numbers immediately before PPI initiation in all PPI users (Figure A-1) and the subgroup of PPI users co-prescribed with antithrombotic (Figure A-2). Figure B assesses the assumption of event-dependent observation. All diagnostic histograms for PPI users (Figure B-1.1), PPI users co-prescribed antithrombotic(Figure B-2.1), and PPI monotherapy users (Figure B-3.1) show a sharp increasing mode for censored plot due to death right after the events.

Figure 1. Diagnostic analysis using visual plots for assessing event-dependence assumptions for standard self-controlled case series


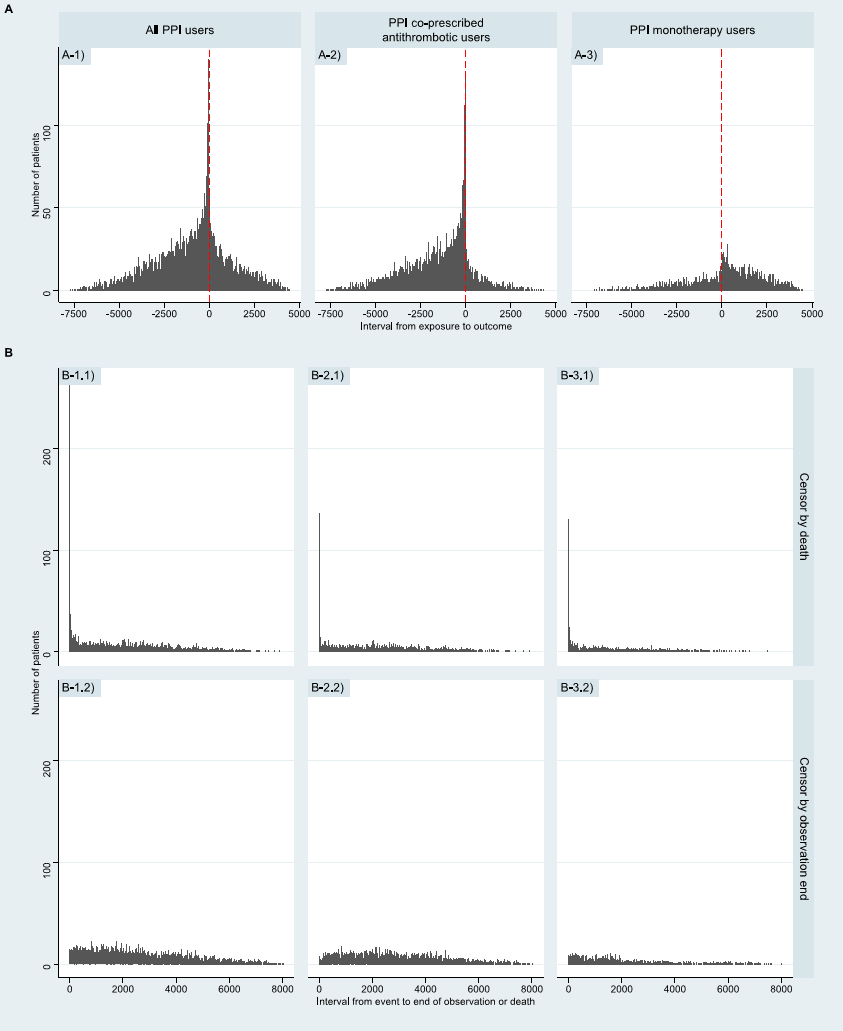


Panel A is for assumptions assessment for event-dependent exposure. Panel A shows the interval distribution from events to exposure in day units. A-1 includes all PPIs participants, while A-2 and A-3 are the stratified analysis for co-prescription with antithrombotic users. In A-1 and A-2, there is increasing pattern right before the Day 0.

Panel B includes figures for assumption check of event-dependent observation. The B-1.1, B-2.1, and B-3.1 shows the number of events against time from stroke to observational end date, with and without censoring at death.

## Supplementary 2. Diagnosis codes and British National Formulary in the analysis

| **Diseases** | **ICD-9-CM code** |
| --- | --- |
| Ischaemic stroke | 433.01,433.11,433.21,433.31,433.81,433.91,434,436,437.0,437.1 |
| Cardiovascular disease | 398.91, 401-405, 410-412, 425.4, 425.5, 425.7-425.9, 427.3, 428, 433, 434, 436-438, 453.8 |
| Peripheral vascular disease | 441, 443.9, 785.4 |
| Respiratory disease | 416.8, 416.9, 490-496, 500-505, 506.4, 508.1, 508.8 |
| Chronic obstructive pulmonary disease | 490-496, 500-505, 506.4 |
| Paralysis | 342, 344.1 |
| Diabetes mellitus | 250 |
| Chronic kidney disease | 582, 585, 586, 588, 583.0-583.2, 583.4, 583.6, 583.7 |
| Mild liver disease | 571.2, 571.4-571.6 |
| Moderate-severe liver disease | 456.0-456.2, 572.2-572.4. 572.8 |
| Ulcers | 531-534 |
| Rheumatoid arthritis and other Inflammatory polyarthropathies | 710.0, 710.1, 710.4, 714.0, 714.1, 714.2, 714.81, 725 |
| Malignancy | 140-149, 150-159, 180-189, 170-172, 174, 175, 176, 179, 160-165, 190-195, 200-208 |
| Metastatic solid tumour | 196-199 |
| **Drugs** | **British National Formulary** |
| PPI | 1.3.5 |
| Oral anticoagulant | 2.8.2 |
| Antiplatelet | 2.9 |

## Supplementary 3. Frequency table for type of PPI drugs in the database

| **PPIs** | **Count (%)** |
| --- | --- |
| Pantoprazole | 297340 (56.68%) |
| Esomeprazole | 102968 (19.63%) |
| Rabeprazole | 88343 (16.84%) |
| Lansoprazole | 29829 (5.69%) |
| Omeprazole | 4702 (0.90%) |
| Dexlansoprazole | 1399 (0.27%) |

## Supplementary 4. Demographic information on the first day of observation for the patients included

| **Demographics** | **All PPI users (%)**  **N = 8170** |
| --- | --- |
|  |  |
| Age, years [mean (SD)] | 65.45 (12.83) |
| Sex, male (%) | 3965 (48.5) |
| Comorbidities |  |
| Charlson Comorbidity Index (%) | 2857 (35.0) |
| Hypertension (%) | 1612 (19.7) |
| Diabetes mellitus (%) | 850 (10.4) |
| Cardiovascular disease (%) | 2673 (32.7) |
| Peripheral vascular disease (%) | 40 (0.5) |
| Respiratory disease (%) | 218 (2.7) |
| Chronic obstructive pulmonary disease (%) | 218 (2.7) |
| Paralysis (%) | 305 (3.7) |
| Chronic kidney disease (%) | 83 (1.0) |
| Mild liver disease (%) | 22 (0.3) |
| Moderate-severe liver disease (%) | 1 (0.0) |
| Ulcers (%) | 268 (3.3) |
| Rheumatoid arthritis and other Inflammatory polyarthropathies (%) | 46 (0.6) |
| Malignancy (%) | 272 (3.3) |
| Metastatic solid tumour (%) | 9 (0.1) |
| Antithrombotic use |  |
| Oral anticoagulants | 57 (0.7) |
| Antiplatelets | 4936 (60.4) |
| Both oral anticoagulants and antiplatelets | 602 (7.4) |
| No antithrombotic | 2575 (31.5) |
| *The past history status was identified on the observation starting day |  |

References

1. Petersen I, Douglas I, Whitaker H. Self controlled case series methods: an alternative to standard epidemiological study designs. BMJ. 2016;354:i4515.

2. Farrington P, Whitaker H, Ghebremichael-Weldeselassie Y. Self-Controlled Case Series Studies: A Modelling Guide with R (1st ed.): Chapman and Hall/CRC.; 2018.

3. Whitaker HJ, Ghebremichael-Weldeselassie Y, Douglas IJ, Smeeth L, Farrington CP. Investigating the assumptions of the self-controlled case series method. Stat Med. 2018;37(4):643-58.
